# Profile and development of adaptive behavior in adults with autism spectrum disorder and severe intellectual disability

**DOI:** 10.3389/fpsyt.2024.1470466

**Published:** 2025-01-08

**Authors:** Jean-Louis Adrien, Romuald Blanc, Eric Thiébaut

**Affiliations:** ^1^ Laboratory of Psychopathology and Health Processes (UR4057), Institute of Psychology, University Paris City, Paris, France; ^2^ Laboratoire Lorrain de Psychologie et Neurosciences de la Dynamique des Comportements, Université de Lorraine, Nancy, Lorraine, France

**Keywords:** autism spectrum disorder, severe intellectual disability, Vineland-II, profiles of socio-adaptive development, heterogeneity, autonomy, Socio-Emotional and Cognitive Evaluation Battery-Adult - SCEB-A

## Abstract

**Background:**

This study examined the profiles of adaptive behavior development in adults with autism spectrum disorder (ASD) and severe intellectual disability (ID), and the relationships between the levels of the different domains and subdomains of adaptive development and the intensity of autistic symptomatology.

**Participants:**

This study involved 71 adults (44 men and 27 women with average ages of 39 years 7 months and 36 years 2 months, respectively) living in medico-social institutions and having a level of adaptive development corresponding to age below 3 years 4 months and a level of cognitive development corresponding to ages between 12 and 24 months.

**Methods:**

ASD was diagnosed using Pervasive Development Disorder-Mental Retardation Scale (PDD-MRS) and Childhood Autistic Rating Scale (CARS), ID and its severity were determined based on the Diagnostic Statistical Manual-5 (DSM-5) criteria, and the very low cognitive developmental level was assessed using the Socio-emotional Cognitive Evaluation Battery (Adrien, Pearson-ECPA, 2007), adapted for adults (SCEB-A). Adaptive development was assessed using the Vineland-II tool.

**Results:**

The adaptive developmental profile was heterogeneous: the median levels of the three domains of socio-adaptive development differed significantly from each other, and the domain of Daily Living was the most developed. Among the nine subdomains, the weakest developmental levels corresponded to Expressive Language, Interpersonal Relationships, and Play/Leisure. By contrast, the highest levels corresponded to Personal and Domestic Autonomy. Significant and negative relationships were noted between the intensity of autism severity and the levels of development in Communication and Daily Living but not Socialization. These results are discussed to highlight the best-developed adaptive domains and those to be improved.

## 1 Introduction

Severe autism spectrum disorder (ASD) is noticeable very early in life, occurs throughout life (1), and necessitates interventions and support, including regular parental support, for the affected individual (2–5). In ASD, clinical heterogeneity is observed at the genetic, neurological, behavioral, and developmental levels (6), and comorbidities often explain the differences in the treatment, disorder course, and outcome of people with ASD. Intellectual disability (ID) is one of the comorbidities that most influence the outcomes of people with ASD, as it can increase autistic symptomatology (7, 8). In a recent study, Etyemez et al. (9) showed evidence that children with ASD with ID, as compared with children with ASD without ID, were more likely to have histories of non-specific developmental delays and neurological disorders. In cases of dual diagnosis, affected children and adults require extensive support and intervention in their daily lives with regard to their particularities. Recently, the concept of “profound autism” was introduced, which refers to several neurodevelopmental disorders and medical conditions in a subgroup of people with ASD, particularly adults, highlighting that these patients have lifelong, intensive support needs (10). Clarke et al. (11) found that the prevalence of profound autism ranged from 11% to 48% among international samples, and this clinical population may be stigmatized. Nevertheless, although a functional classification for autism in adults was devised to distinguish among the different degrees of dysfunction for research and clinical practice (12), the concept of profound autism was considered by Kapp (13) to be limited because it may include people with severe ID and numerous autistic behaviors but without ASD diagnosis, promoting the segregation of this population and preventing their adequate treatment.

Numerous adults with severely handicapped communication and cognitive abilities, as well as health issues (and exhibiting both ASD and severe ID, which classify them as having profound autism), are sent to live in specialized institutions for a long time, sometimes since childhood or adolescence, so they can benefit from intensive and daily interventions (10). However, a literature review revealed that there are only a few clinical studies involving this population, particularly regarding their adaptive functioning in terms of developmental approach.

Adaptive functioning refers to abilities that are essential for carrying out everyday tasks (e.g., preparing meals, dressing, shopping, grooming, and using transportation), communicating with others (e.g., expressing needs through language, writing to convey information, and making phone calls to exchange information), and maintaining a social life (e.g., interpersonal exchanges, establishing emotional relationships, and living in a group while respecting the rules of collective life). The Vineland Adaptive Behavior Scale (VABS), particularly the validated second version developed in France, the Vineland-II, is the recommended tool for assessing adaptive functioning (14–16). Most studies that have used this tool on individuals with ASD to evaluate adaptive functioning have focused primarily on understanding the discrepancies between the levels of intellectual ability and adaptive development (17–19), as well as the link between adaptive development, ID comorbidities, and other psychiatric and/or neurophysiological disorders (20) and their functional outcomes (21). In addition, Chatham et al. (22) demonstrated that Vineland-II highlights minimum clinically important differences (MCIDs) in people with ASD, observed during the disorder course or under treatment. These studies have shown that communication skills, daily autonomy, and socialization skills are generally less developed than intellectual ability [intelligence quotient (IQ)] among people with ASD regardless of age. Increased intellectual abilities are associated with a slight increase in coping skills (17, 23), whereas auditory processing disorder is correlated with social inadaptation behaviors (20). Other studies have focused on profiles of adaptive behavior skills in people with ASD, with or without ID (24, 25), highlighting the negative effect of ID and other psychopathologies on adaptive functioning. Moreover, Tillmann et al. (26) found evidence that people with lower IQ, older age, and more impaired social communication abilities had lower adaptive functioning.

All these studies have provided evidence for the value of assessing adaptive behavior for intervention and treatment in people with ASD, accounting for ID severity. However, no study has focused on adults with both ASD and very low cognitive developmental levels corresponding to a severe or profound ID (IQ < 20) or described the profile of adaptive development and its relationship with the low level of cognitive and socio-emotional development or the intensity of autistic symptomatology.

Therefore, this work aimed to characterize the socio-adaptive developmental profiles of adults with ASD, associated with severe ID. The chosen population had cognitive and socio-emotional levels equivalent to those of children under 2 years and adaptive levels equivalent to those under 3 years. Moreover, this study aimed to determine whether the level of adaptive development differed from that of cognitive and socio-emotional development and whether the adaptive developmental profile was consistent in identifying domains with low and high developmental levels and to examine the relationships between the levels of development of the different socio-adaptive domains and subdomains and the severity of autistic symptomatology.

## 2 Materials and methods

### 2.1 Participants

This study involved 71 adults with ASD and a very low cognitive developmental level, evidencing severe ID [44 men and 27 women, with average ages of 39 years 7 months and 36 years 2 months, respectively (minimum = 18 years; maximum = 67.5 years)]. These 71 individuals were selected from a group of 90 adults who participated in the research. The participants were recruited during 2022 and 2023 from 16 clinical services specializing in care and education for adults with ASD and severe ID. These clinical services included specialized reception homes (SRHs), which were established to help disabled people in situations of high dependency to lead independent lives and benefit from care adapted to their needs. The disabled individuals (about 30 adults in each of these) stay at the SRH during the week and can go home during the weekend and some holidays. The French National Authority for Health (4, 5) states that, in the SRH, each adult must benefit from an individualized program elaborated in coordination and agreement with both professionals and the individual’s parents (or family). The program may include personal and domestic autonomy (e.g., meal preparation, linen storage, dishwashing, and cleaning of common living rooms), recreational activities (e.g., walking, watching TV, listening to music, and physical activities like horse riding), and training to develop social abilities such as verbal and non-verbal expressive and receptive language education with a speech therapist, as well as personalized medical care. All the clinical services that were considered in this study followed these recommendations. Notably, the participants lived in other weekday care services during childhood and adolescence given the severity of their disorders and moved to SRHs when they reached adulthood. At these institutions, the participants were coached by special needs educators and cared for by health professionals with diverse specializations depending on the participants’ health needs (e.g., physicians, psychiatrists, clinical geneticists, and neurologists). These health professionals worked either at the institution or in the nearest hospital. There were some adults with ASD and a very low cognitive developmental level, indicating severe ID, among the selected individuals living in these 16 institutions.

The inclusion criteria were ASD and very low global cognitive developmental levels, with the latter characterized by a cognitive developmental level inferior to 2 years of age and a socio-adaptive level inferior or equal to 3 years of age. The mentioned services partnered with Paris City University (cf. list of associations and medico-social services and partner psychologists). Information and consent forms were given to participants and their legal guardians. The study was conducted in accordance with official laws and standards of ethics of biomedical and clinical research in France; it received approval from the research ethics committee of Paris City University (No. 2021-42).

#### 2.1.1 Diagnosis of ASD in adults with low cognitive level

The diagnosis of ASD was made using the DSM-5 criteria (27) such as difficulties in social communication and interactions and some restrictive, repetitive behaviors or interests with atypical sensorial reactivity. As demonstrated by Thurm et al. (28), an ASD diagnosis is problematic in adults with a very low cognitive developmental level because they cannot use verbal or non-verbal communication. Indeed, it is difficult to differentiate ASD from severe or profound ID, particularly among those who exhibit physical disabilities, sensory deficits, and/or genetic conditions, that is, among those who suffer from profound intellectual and multiple disabilities (PIMD). Thus, the diagnostic process should be enhanced for people with PIMD to better understand the genetic architecture of various ASD genetic subtypes (29). For example, Oberman et al. (30) found that a general developmental delay significantly contributed to the ASD diagnosis in people with Phelan–McDermid syndrome and moderate ID. There are hardly any diagnostic tools adapted to the clinical population of the present study. However, Diagnostic Behavioral Assessment for Autism Spectrum Disorder-revised (DIBAS-R) (31–33), which was constructed and validated for screening ASD in adults with ID, may be a highly relevant diagnostic tool. Nonetheless, it has not been adapted to or validated in the French population. Moreover, while recent modifications of the Autism Diagnostic Observation Schedule-2 (ADOS-2) are appropriate for minimally verbal and older individuals with a non-verbal mental age of at least 18 months (34), this version has not been adapted to a French sample. Furthermore, some of the adults included in the present study had non-verbal ages inferior to 18 months. Therefore, we used PDD-MRS (35, 36), a quantitative diagnostic assessment tool for ASD, validated in a French clinical population and generally used to diagnose ASD in adults with mild, moderate, or severe ID. In addition, we used CARS to determine the degree of severity of the autistic symptomatology and thus confirm the ASD diagnosis of these adults (37). While the first version of CARS was equivalent to the second (CARS2-ST), Ji et al. (38) demonstrated the concurrent validity of CARS2-ST by its significant correlation with ADOS-2 in a sample of 237 children (aged 24–145 months). Moreover, Dawkins et al. (39) found that CARS2 resulted in a high diagnostic agreement with the DSM-IV and DSM-5 criteria for autism. Even though these results concern only children, we may consider that the association between CARS and PDD-MRS is relevant to confirm the ASD diagnosis in our study’s participants.

The PDD-MRS was administered by the psychologist or educator who knew about the adult’s behaviors and functioning in daily life and who was responsible for their individualized program. This tool produced a score between 0 and 19, with a minimum cut-off score of 10 indicating ASD. CARS scores range from 0 to 60, with a minimum autism cut-off score of 30. Hierarchical score classes indicate mild, moderate, and severe autism. Assessment using the PDD-MRS was performed on 44 of 71 adults. All adults in this subgroup were diagnosed with ASD, using the PDD-MRS, except for two who had PDD-MRS scores between 0 and 6. However, these two adults exhibited autistic symptomatology based on their CARS scores, which were superior to the cut-off scores (36.5 and 32.5). Assessment using CARS was performed on 67 adults (four had data missing). The mean score was 44.6 (SD = 5.95, min. = 32, max. = 57.5), indicating severe autism.

#### 2.1.2 Identification process of the very low cognitive developmental level of adults implying ID diagnosis

Because the adults in this study exhibited very low cognitive development relative to their chronological age, we may conclude that they suffered from ID. However, according to DSM-5, the ID diagnosis should be performed with intelligence tests that yield standard scores such as IQ, ranking the individual in their chronological age group. The degree of ID severity is determined by this rank, which corresponds to at least one standard deviation below the mean of the individual’s age group. Most intelligence or cognitive tests were developed to assess adults with severe, moderate, or mild ID. For instance, WAIS-IV (40) is used for people with chronological ages from 16 to 79 years 11 months. Some tests like Wechsler Nonverbal Scale of Ability (WNV) (41, 42) can be used to assess children, adolescents, and adults from 4 to 21 years 11 months. However, there are no standardized tests adapted to a French population to assess cognitive functions in adults with very severe cognitive disabilities, dysfunction, and delays and who may be considered to exhibit profound ID. Indeed, psychologists have noted that such individuals cannot respond to simple verbal instructions, name words, or perform simple non-verbal tasks in standardized tests such as WAIS-IV. Thus, it is virtually impossible to get such individuals to complete standardized tasks, and they are considered unfit to take standardized tests. Thus, decisions relative to their care and education are generally based on their behaviors and medical, cognitive, and adaptive disorders. This problem is accentuated when the adult exhibits an ASD. Although Heinrich et al. (31) used the Disability Assessment Schedule (DAS) (43, 44) to validate the DIBAS-R of adults with ASD and mild, moderate, severe, and profound ID, this schedule has not been validated in France.

For the abovementioned reasons, in the present study, no adults with ASD could be assessed using standardized intelligence tests. Thus, to solve this problem and help clinicians identify adults with severe IDs, we first asked the psychologists and educators of each SRH to complete an original clinical scale, allowing us to rate the adults’ ability level in each of both domains (Social and Personal Autonomy, and Communication). They had to rate the absence (score of 0) or presence (scores of 1 and 2) of the adults’ social and personal autonomy behaviors and their communication ability with expressive language or gestures, empirically hierarchized by developmental levels (scores 0, 1, 2, 3, and 4). The total minimum and maximum scores were 0 and 7, respectively. Based on the score, clinicians assigned the developmental level and limitations of each adult and indicated the degree of severity of the ID: a moderate ID was defined as 30 < IQ < 50 (scores 3–7), and a severe or profound ID was defined as IQ < 30 (scores 0–2).

All the adults selected for this study were considered to have a severe or profound ID, based on the clinical judgment by psychologists and psychiatrists instead of standardized test results for the reasons described above. Then, to precisely determine the cognitive developmental level of each adult, their cognitive and socio-emotional development was evaluated using Socio-emotional Cognitive Evaluation Battery (SCEB)-A (see below). Those among the group of 90 adults who were clinically diagnosed as having a moderate ID were not included in this study.

Among the participants, some individuals were affected by various somatic and/or genetic disorders [epilepsy, *n* = 14; Down syndrome, *n* = 3 (one of them also had epilepsy); Sotos syndrome, *n* = 1; Dravet syndrome, *n* = 1; Pierre Robin syndrome, *n* = 1; 22q11.2 deletion syndrome, *n* = 1], which were associated with behavioral (aggressiveness, *n* = 11) and mental (anxiety, *n* = 25; depression, *n* = 3) comorbidities.

#### 2.1.3 Cognitive and socio-emotional developmental assessment

To assess the cognitive developmental level of the 71 selected adults with ASD, we used the SCEB, initially created for children and adolescents with ASD and developmental ages between 4 and 24 months (45, 46). The developmental validity of SCEB was demonstrated based on the high correlations between the overall scores of the SCEB and the psychomotor developmental ages, calculated using the Brunet–Lézine Revised Psychomotor Development Scale (47), a French adaptation of Gesell’s developmental scale (48), which includes assessments of the postural, language, oculomotor coordination, and sociability domains for chronological ages from 1 to 30 months. Recently, a study explored the theoretical and empirical developmental sensitivity of SCEB items for assessing young (typically developing) children and showed evidence of very good hierarchization in four developmental levels, corresponding to ages, for each domain (49).

The SCEB was recommended by the French National Authority for Health (4, 5) for the examination of French autistic children and was used for several years in various studies implying preschool children and children with ASD and moderate or slight ID (50–53), children with ASD and ID with different chronological ages and developmental quotients (40), from different nationalities and countries (51, 52, 54, 55), and children with ASD and ID in the context of genetic syndromes (56–58); recently, it has been adapted to adults (SCEB-A) with very low developmental levels (59–61). The battery includes 16 scales divided into two areas, cognitive and socio-emotional. The cognitive area includes seven scales: self-image, symbolic play, schemata relationship to objects, operational causality, means/ends, spatial relationships, and object permanence. The socio-emotional area includes nine scales: behavior regulation, social interaction, joint attention, expressive and receptive language, vocal and gestural imitation, affective relationship, and emotional expression. Each scale is composed of hierarchical items that determine four cognitive and socio-emotional developmental levels. Level 1 corresponds to the age range of 4–8 months; Level 2, 8–12 months; Level 3, 12–18 months; and Level 4, 18–24 months (37). When Level 1 was not reached, a score of 0 was given. The level score for overall development is the average of the 16-domain level [Global Development Level (GDL)] scores, the cognitive development score is the average of the seven-domain scores [Cognitive Development Level (CDL)], and the socio-emotional development score is the average of the nine-domain scores [Social-Emotional Development Level (SDL)]. A median score can also be calculated. Heterogeneity indexes of the profiles for overall, cognitive, and socio-emotional functions were also calculated. These indexes corresponded to the means of differences (absolute value) between all the level scores (0 to 4) of each of the 16 domains, multiplied by 10 (51, 52). They ranged from 0 (no heterogeneity) to 21 (maximum heterogeneity).

Psychologists trained in using SCEB-A assessed the participants. Each adult was assessed by the psychologist employed at each of the 16 institutions. The adult was accompanied by his/her educator, and the examination was conducted in a dedicated room, considering the individual’s attention, availability, and fatigue. The SCEB-A material was progressively proposed to the adult, who was invited to manipulate it and respond to the psychologist’s requests, using non-verbal interaction, vocal and gestural imitation, and joint attention behaviors or employing objects in a functional or symbolic way. When the adult no longer wanted to participate or could not be attentive, the psychologist stopped the examination and scheduled a second session. The objective was to complete all the items of the SCEB-A protocol according to the adult’s availability.

### 2.2 Measurement of socio-adaptive development

Socio-adaptive development was assessed using the second version of the VABS, VABS-II (14, 15), which was completed by the psychologist and educator responsible for the individualized program of the adults. The VABS-II is a hetero-questionnaire that includes 433 items exploring four domains organized into nine subdomains: Communication (Receptive, Expressive, and Written), Daily Living (Personal, Domestic, and Community), Socialization (Interpersonal Relationships, Adaptation, and Play/Leisure), and Motor Skills (Gross and Fine). Motor skills are only assessed if the person’s chronological age is less than 7 years. VABS-II has been used in France for people from 1 to 90 years of chronological age. After the assessment, the raw scores corresponding to the sum of the item scores in each subdomain are calculated. These raw scores can be used to obtain the scale scores of each subdomain in a specific norm table in the VABS-II manual. The sum of the scale scores of the three subdomains in each domain is used to obtain the standard note in another norm table in the VABS-II manual. The standard note and scale scores indicate the individual’s rank in his/her chronological age group. From the raw scores of each subdomain, we can obtain the developmental age equivalent (DAE), which varies in the French adaptation from <1 year to 18 years or more (see the tables of the VABS-II manual). DAE was calculated based on the sample and corresponds to the mean chronological age (in years and months) at which the sample’s population has obtained it. In the US version, DAEs vary from 1 month to 18 years or more. The US version allows for the determination of DAEs between 1 and 12 months, but the French adaptation does not. Thus, in this study, which used the French version; when the raw score corresponded to <1 year, we attributed a DAE of 8 months to perform the statistical analysis.

Therefore, we used the standard and scale scores obtained by the participants in the subdomains and domains, and the participants’ DAEs.

## 3 Results

The scores produced for the study are ordinal, and the distributions do not appear Gaussian. Consequently, we applied non-parametric tests with the Friedman test, followed by pairwise comparisons (59, 62), which is a non-parametric alternative to analysis of variance adapted to repeated measures).

### 3.1 Cognitive and socio-emotional development

**Table 1** presents the developmental, cognitive, and socio-emotional characteristics of the 71 studied adults.

**Table 1:** Values of mean (M), median (Md), standard deviation (SD), interquartile range (IQR), minimum and maximum SCEB-A development scores levels in global developmental (GDL), cognitive development area (CDL) and its seven domains, and socio-emotional development area (SDL) and its nine domains of the 71 adults.

Areas and domains	M	Md	SD	IQR	Min	Max
SCEB-A—Global Development Level (GDL)	2.5	2.7	0.7	1.0	0.7	4.0
SCEB-A—Social-Emotional Development Level (SDL)	2.4	2.3	0.7	0.9	1.0	4.0
1. Behavior regulation	3.5	4	0.8	1.0	1	4
2. Social interaction	2.3	2	0.9	1.0	1	4
3. Joint attention	3.2	4	1.2	2.0	0	4
4. Expressive language	1.5	1	1.5	3.0	0	4
5. Receptive language	3.1	3	1.1	1.0	0	4
6. Vocal imitation	0.9	0	1.3	1.0	0	4
7. Gestural imitation	1.6	2	1.5	2.0	0	4
8. Affective relationship	3.4	4	1.0	1.0	0	4
9. Emotional expression	2.5	3	1.3	3.0	0	4
SCEB-A—Cognitive Development Level (CDL)	2.6	2.9	0.9	1.4	0.3	4.0
1. Self-image	2.3	2	1.4	3.0	0	4
2. Symbolic play	1.9	2	1.1	2.0	0	4
3. Schemata relationship to objects	2.9	3	0.9	0.0	0	4
4. Operational causality	2.3	3	1.5	3.0	0	4
5. Means–ends	2.9	3	1.1	2.0	0	4
6. Spatial relationship	3.5	4	1.0	0.5	1	4
7. Object permanence	2.3	2	1.4	2.5	0	4

Median (Md) levels values of each domain and chronological period of ages’ correspondence in young children: 0 = aged <4 months, 1 = aged 4–7 months 29 days, 2 = aged 8–11 months 29 days, 3 = aged 12–17 months 29 days, and 4 = aged 18–24 months.In gray: Global, socio-emotional and cognitive development values.

The median (Md) level values of each domain and chronological period of ages’ correspondence in young children are as follows: 0 = aged <4 months, 1 = aged 4–7 months 29 days, 2 = aged 8–11 months 29 days, 3 = aged 12–17 months 29 days, and 4 = aged 18–24 months.

**Figure 1:**
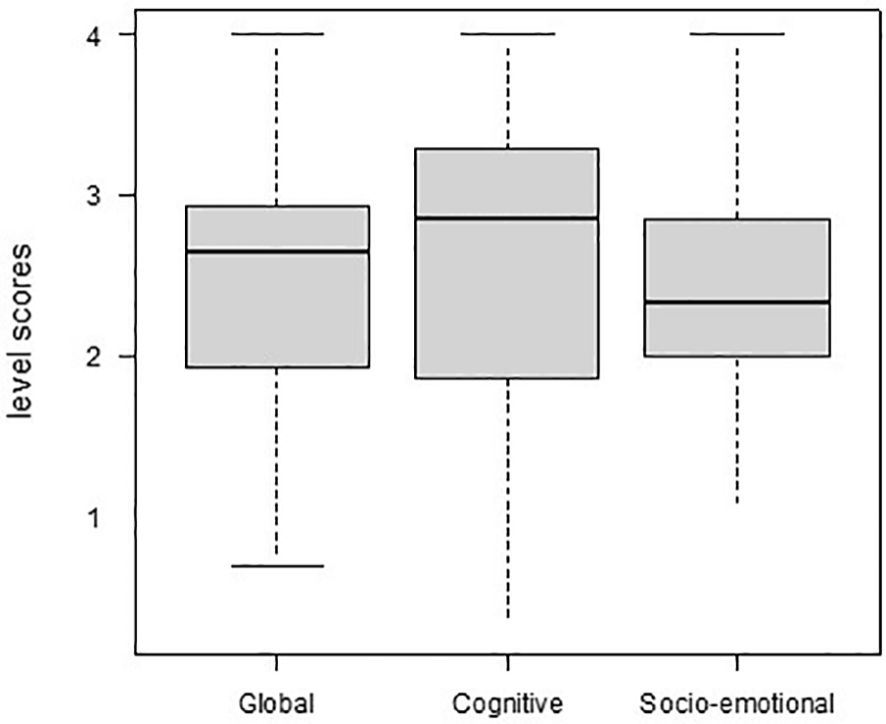
Median scores level in global, cognitive, and socio-emotional development from Socio-emotional Cognitive Evaluation Battery (SCEB)-A of the 71 adults.

Note: Kendall’s W was used as a measure of effect size for Friedman’s test (Tomczak & Tomczak, 2014). It uses Cohen’s interpretation recommendations: 0.1 to < 0.3 (small effect), 0.3 to < 0.5 (moderate effect), and ≥0.5 (large effect).

The median global, cognitive, and socio-emotional development scores of the 71 adults were 2.65, 2.85, and 2.33, respectively, corresponding to the developmental age of 12–15 months. There was a significant difference [*χ* ^2^ Friedman (1, *N* = 71) = 4.63, *p* = .031, w_*_ = 0.07] between the median cognitive and socio-emotional levels (**Figure 1**), indicating that the developmental level was lower in communication, language, and imitation than in cognition.

### 3.2 Adaptive functioning developmental profile

All the calculated scale scores of the VABS-II subdomains in this study were between 1 and 7, and the standard notes of the domains were between 20 and 29 (**Table 2**). For each of the nine subdomains, the 69 adults had minimum scale scores of 1 (percentile < 1), except for one adult’s receptive language subdomain (score = 6), one adult’s written language subdomain (score = 3), and two adults’ personal autonomy subdomain (scores = 3 and 2). Furthermore, in the domestic autonomy subdomain, one adult scored 7, two adults scored 6, two adults scored 5, one adult scored 4, and two adults scored 2. These low standard and scale scores may be explained by the fact that the evaluated adults had very low socio-adaptive developmental levels (DAE) relative to their chronological ages (18–67.3 years). Thus, the gap between the highest standard or scale scores and the lowest scores was too small to perform differential statistical analysis permitting to show evidence precisely of their socio-adaptive profile and substantial differences between the three domains or the nine subdomains of VABS-II. However, the calculated DAE scores ranged from 8 to 127 months of age (**Table 2**). Therefore, because this work was concerned with adaptive development, only DAE values were considered for the statistical analysis, excepted for Writing not interpretable in this study. Moreover, as the VABS-II norms do not indicate DAEs for Communication, Daily Living, and Socialization domains, the DAEs calculated corresponded to the median and mean values of their three subdomains (only two for Communication: Receptive and Expressive). The global DAE corresponded to the mean and median of all three domains. This DAE choice was mainly determined by the objective to focus on the developmental levels of adaptive functioning abilities and not to identify the degree of severity of socio-adaptive handicap such as those noted by standard and scale scores.

**Table 2:** Values of mean (M), median (Md), Standard Deviation (SD), interquartile range (IQR), minimum and maximum of standard scores of the 3 domains and scale scores of the 8 subdomains of the 69 adults (VABS-II).

	M	Md	SD	IQR	Min	Max
VABS-II – Standard score Communication	20	20	0	0	20	20
1. Scale score Receptive	1.1	1	.6	0	1	6
2. Scale score Expressive	1	1	0	0	1	1
VABS-II - Standard score Daily Living	20.2	20	1.3	0	20	29
1. Scale score Personal Autonomy	1.04	1	.27	0	1	3
2. Scale score Domestic Autonomy	1.6	1	1.3	1	1	7
3. Scale score Community Autonomy	1	1	0	0	1	1
VABS-II - Standard score Socialization	20	20	0	0	20	20
1. Scale score Interpersonal Relationships	1	1	0	0	1	1
2. Scale score Play/Leisure	1	1	0	0	1	1
3. Scale score Adaptation	1	1	0	0	1	1
VABS-II – Global DAE	19.5	17.6	8.2	11,8	8.4	42.9
VABS-II - DAE Communication	14.4	12.5	6.9	5	8	50
1. DAE Receptive	15.9	16.0	7.0	6.0	8.0	39
2. DAE Expressive	12.9	10.0	8.7	6.0	7.0	61
VABS-II - DAE Daily Living	31.1	27.0	16.6	24.7	8.0	70.7
1. DAE Personal Autonomy	33.3	31.0	17.1	21.0	8.0	107
2. DAE Domestic Autonomy	40.9	27.0	32.4	50.0	5.9	127
3. DAE Community Autonomy	19.2	12.0	12.6	21.0	8.0	57
VABS-II - DAE Socialization	12.3	11.0	4.9	4.3	4.3	25
1. DAE Interpersonal Relationships	10.2	8.0	4.1	2.0	3.0	27
2. DAE Play/Leisure	11.6	8.0	6.8	4.0	8.0	41
3. DAE Adaptation	16.1	13.0	6.9	8.0	8.0	37

Values of the Development ages equivalent (DAE) of 3 domains of adaptive functioning and the 8 subdomains of the 69 adults (VABS-II). VABS, Vineland Adaptive Behavior Scale. In gray: Standard and DAE scores of three domain.

### 3.3 Adaptive functioning profile by domains and subdomains

The values of standard scores, scale scores and DAE of three domains of adaptive functioning and the eight subdomains of 69 adults are presented in **Table 2** (data were missing for two participants).

#### 3.3.1 The three adaptive functioning domains

The mean values of the mean and median DAEs of all three adaptive domains were respectively 21.1 months and 18.4 months.

**Figure 2:**
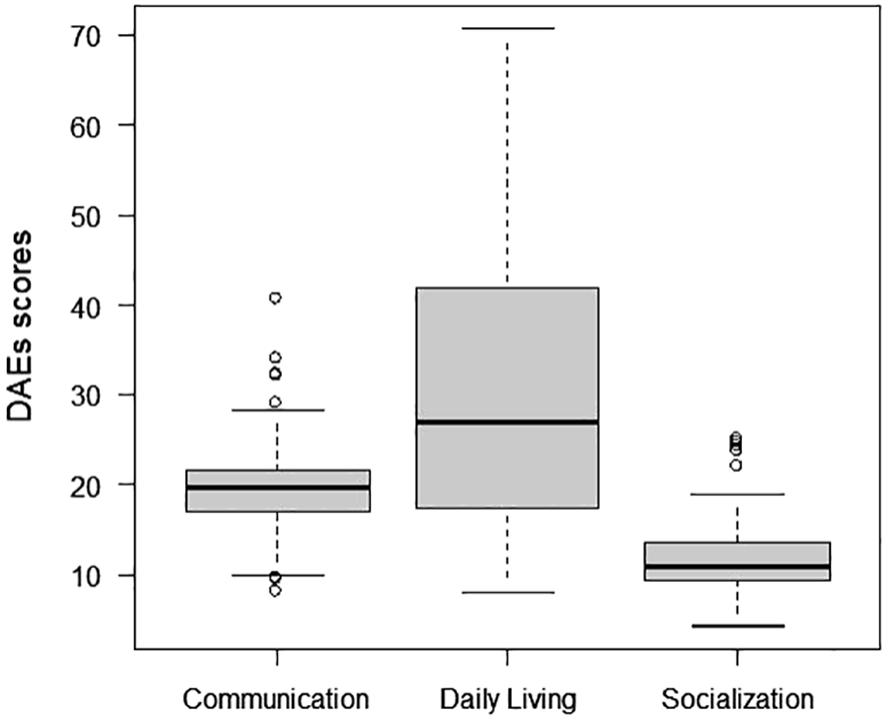
Profile of the median development ages equivalent (DAE) and interquartile range of the three domains of socio-adaptive development.

**Figure 2** presents the mean and median values of the median DAE of three socio-adaptive domains of the 71 adults.

Comparisons of the DAEs for Communication, Daily Living, and Socialization were performed using nonparametric tests suitable for repeated measurements (*χ* ^2^ Friedman (2, *N* = 69) = 101, *p* <.001, *w* = 0.73). Multiple comparisons were followed by analytical comparisons (34) (**Table 3**).

The DAEs of the three domains were statistically different, with the Daily Living level being the highest and Socialization the lowest.

#### 3.3.2 The eight adaptive domains

Analytical comparisons were made for the “strength/weakness” identification for each of the three socio-adaptive functioning domains.

For Communication (Multiple comparisons: *χ* ^2^ Friedman (2, *N* = 69) = 60.3, *p* <.001, *w* = 0.44) (**Table 3**), there were significant differences between the values of the Expressive and Receptive DAEs with those of receptive subdomain being more developed.

For Daily Living [multiple comparisons: *χ* ^2^ Friedman (2, *N* = 69) = 54.6, *p* <.001, *w* = 0.40] (**Table 2**), there were significant differences only between the subdomain Autonomy in Community and the two other subdomains (Personal Autonomy and Domestic Autonomy) (**Table 3**), with Personal Autonomy being the most developed (DAE = 31 months).

**Table 3:** Testing for pairwise differences in median development ages equivalent of domains and sub-domains scores Vineland-II of the 69 adults.

Pairwise comparison			*χ* ^2^ (Durbin-Conover)	*p*
Three domains of adaptive functioning				
DAE Communication	–	DEA Daily Living	15.3	< .001
DAE Communication	–	DEA Socialization	2.58	.011
DAE Daily Living	–	DEA Socialization	17.88	< .001
DAE Communication subdomains				
Receptive	–	Expressive	4.8	< .001
DAE Daily Living subdomains				
Personal	–	Community	8.06	< .001
Personal	–	Domestic	0.22	0.824
Community	–	Domestic	8.28	< .001
DAE Socialization subdomains				
Interpersonal Relationships	–	Play/Leisure	1.93	0.055
Interpersonal Relationships	–	Adaptation	11.16	< .001
Play/Leisure	–	Adaptation	9.23	< .001

For Socialization [multiple comparisons: *χ* ^2^ Friedman (2, *N* = 69) = 70.6, *p* <.001, *w* = 0.51] (**Table 4**), there were significant differences only between the Adaptation subdomain and the other subdomains (Interpersonal Relations and Play and Free Time), with Adaptation being the most developed (DAE = 13 months).

### 3.4 Relationships between socio-adaptive development and autistic symptomatology

Finally, we investigated whether the intensity of autistic symptomatology of the 67 adults (scores obtained on the CARS) was related (Spearman’s rank correlation) to the overall DAE of socio-adaptive development; the DAEs of Communication, Daily Living, and Socialization; and those of each subdomain (**Table 4**).

**Table 4:** Correlations between CARS scores and different domains, and subdomain scores using Vineland-II of the 67 adults (four had data missing on CARS scores).

Vineland II	CARS (*r* Spearman)	*p*
DAE Global	−.52	<.001
DAE Communication	−.38	.002
DAE Daily Living	−.53	<.001
DAE Socialization	−.17	.172
Receptive	−.43	<.001
Expressive	−.44	<.001
Writing	.29	.025
Personal Autonomy	−.35	.004
Community autonomy	−.44	<.001
Domestic Autonomy	−.52	<.001
Interpersonal Relationships	−.32	.009
Play/Leisure	−.30	.017
Adaptation	−.12	.344

DAE, developmental age equivalent.

The severity of autistic symptomatology was significantly correlated with a low overall socio-adaptive level, a low level of autonomy in daily living (particularly the levels of development of domestic autonomy and autonomy in the community), and a low level of expressive and receptive communication. We noted that the socialization domain level and one of the subdomains, Adaptation, were not correlated to the severity of autism.

## 4 Limitations

Some limitations of this study must be indicated. First, the recruitment method that was empirical and based on spontaneous contacts may introduce selection bias and potentially limit the generalizability of the findings. However, we met the primary and basic inclusion criterion: all adults came from only specialized and medicalized establishments, as defined by French law, that welcome and care for severely disabled people with autism and severe intellectual disability and who also suffer from psychological and medical comorbidities.

Second, although we could have used DIBAS-R (29, 31, 32) or recent modifications of ADOS for older people with ID (33) to screen for ASD in the participants, these tools were not available or validated in France.

Moreover, we could have used a cognitive test such as DAS to measure ID in the participants (42), but again, this test was not adapted to the French context.

We considered standard and scale scores for all subdomains and domains of the VABS-II that indicate the individual’s rank in relation to their chronological age reference group. However, the scores were too similar and did not allow us to perform differential statistical analysis to determine the profiles of the domains and subdomains of socio-adaptive development. The standard or scale scores only determine the degree of severity of the impairment in socio-adaptive abilities, which, in the case of our group of adults, indicate a percentile rank inferior to 1 in all the subdomains and domains. Thus, even if the obtained results could be compared to other studies using standard data and could be generalized, they would not allow us to understand the peculiarities in the adaptive development of this specific population. Therefore, a developmental approach was more relevant in our case because it enabled us to estimate the developmental level for each subdomain and domain. Hence, we used the DAE that corresponded to the acquisition level of the behaviors performed by the individual, indicating the mean chronological ages at which the behaviors were exhibited or acquired in the sample population, calculated from French norms. If other studies intend to replicate this procedure, they could use this type of developmental data, which is available in the original US version of the VABS-II (DAE from birth to 90 years) and should be present in all validated versions for all countries.

Third, the study’s cross-sectional nature limits the ability to infer causal relationships or developmental trajectories over time. Therefore, it would be interesting to assess again some adults of all the groups and study modifications or not of socio-adaptive abilities over time.

Moreover, this study was only focused on adults with ASD and severe ID, and it would be relevant to include a control group such as adults with severe ID without ASD in order to strengthen the comparative analysis of adaptive behaviors and identify specific weaknesses or strengths in the autistic group.

Finally, the study was based on participants from France, and the findings may not be generalizable to other cultural contexts due to differences in care systems and societal expectations. However, if similar methodological strategies are applied, such as the screening of ID and ASD diagnosis and the cognitive and socio-emotional assessment using SCEB-A, replicable studies may be conducted.

## 5 Discussion

The study focused on the socio-adaptive development assessed using the Vineland-II in 71 adults with ASD, severe ID, somatic problems (epilepsy or genetic syndrome), and behavioral difficulties. The participants were recruited from several medico-social institutes in six French regions, and the recruitment was empirically based on spontaneous contacts with various psychologists and psychiatrists who worked in French private or public services and had contact with the participants. We faced some limitations in diagnosing ASD in people with very low overall development (i.e., inferior to 18 months) (28), especially considering that the diagnostic instruments adapted to this clinical group of adults have not been validated in France; however, simultaneously using PDD-MRS and CARS enabled us to consider these adults as exhibiting ASD.

There are no intelligence tests in France adapted to a population with very low cognitive development, associated with various medical, behavioral, and psychological conditions; thus, we could not use standardized tests on our participants. Nevertheless, the assessment of cognitive development using SCEB-A demonstrated the adults’ very low cognitive development because their mean value corresponded to ages 12–15 months, with the minimum being inferior to 4 months and the maximum corresponding to 24 months. These results demonstrated the heterogeneity of the cognitive development of these adults; however, in all cases, they confirmed the participants’ intellectual disabilities.

The standard scores of domains and scale scores of subdomains, obtained by the adults in VABS-II, were very low and demonstrated the participants’ severe adaptive disabilities. Moreover, these values were similar for all participants, implying that a statistical comparative analysis would have been unable to describe the specificities of the adaptive profile or identify differences between the developmental levels corresponding to the various domains and subdomains. Therefore, the statistical analysis was mainly focused on the DAE, whose minimum and maximum values were 8 months and 127 months of age, respectively.

Results show evidence the median of the global DAE of socio-adaptive development (17.6 months) was slightly higher than that of cognitive and socio-emotional development (12 to 15 months), contrary to what was noted previously (17, 18). Thus, our results show that adults with ASD and severe ID can develop better behavioral adaptive functioning than cognitive functioning, which may be explained by the stagnation of their cognitive development and the constant and long-lasting education in adaptive behaviors that benefit them and allow them to make elementary progress in daily life. This result may show that “potential changes” in adults with profound autism such as those noted by Lord et al. (10) were possible over time even though they can be minimal (22). However, to confirm and validate it, it would be necessary to perform longitudinal studies.

Furthermore, high heterogeneity was observed in the socio-adaptive profiles of the adults in the study at both the inter-domain and inter-individual levels. The median DAEs in the three domains of socio-adaptive development of these adults were as follows: the DAE of Communication was 12.5 months, the DAE of Daily Living was 27 months, and the DAE of Socialization was 11 months. Their daily adaptation to a stable and structured environment was at a higher developmental level than their communication or socialization skills, the latter of which remained particularly precarious. The results also revealed a strong disparity in these three socio-adaptive domains.

A more detailed analysis of the skills of these adults revealed several key points: they showed higher abilities in receptive than in expressive communication, they had a higher level of development in personal autonomy than in domestic autonomy, and their level of autonomy within their social group appeared to be more deficient. Finally, their socialization skills were lacking in terms of interpersonal relationships and the management of play and leisure. However, they showed more social adaptation skills with familiar people. Thus, these results show that adults with ASD and severe ID always have more difficulty developing communication and social skills over time than learning daily life activities. Although the mean global cognitive and socio-emotional developmental level was low (between 12 to 15 months of age), the adults with higher DAEs could develop personal and domestic autonomy (33.3 and 40.9 months, respectively).

In addition, the severity of the participants’ autistic symptomatology appeared to be strongly correlated with their low levels of overall social adaptation, expressive language development, and autonomy in daily life, particularly domestic and community autonomy. This confirms that the more autistic and very low verbal levels adults have the most difficulty carrying out activities that require abilities in contact and exchange with other people. However, we noted that the socialization developmental level was not related to the severity of autism, indicating that all these adults—who showed a very low socialization developmental level (min. = 8 months; max. = 25 months)—exhibited different degrees of severity in their autistic symptomatology (min. = 32; max. = 57.5). This finding suggests that adults with ASD and severe ID, whatever the degree of severity of their autistic symptomatology, cannot perform even elementary socialization actions, confirming their severe social handicap.

This study highlights the importance not only of performing regular assessments of adults with ASD and severe ID in the socio-emotional, cognitive, and adaptive areas to identify their profiles and update their individualized intervention programs accordingly but also of recognizing and supporting socio-adaptive behaviors in this population, such as socialization. At last, note that the cognitive and socio-emotional developmental levels were lower than adaptive functioning in these adults with ASD and severe ID, showing the need to always stimulate their cognitive and communicative functions.

## 6 Studies in perspectives

### 6.1 Increase the performance of diagnostic and developmental tests for adults with ASD, very low overall development, and severe ID

This is the first study in France concerning the adaptive development of a clinical population of adults with ASD and severe ID, associated with various medical comorbidities such as genetic syndromes, epilepsy, and behavioral and mental comorbidities including aggressiveness and anxiety. Thus, future complementary studies could be conducted to expand on our findings. Here, we had to enhance the performance of diagnostic assessments for both ASD and ID to better understand and differentiate the adaptive profiles of the participants according to the severity of ASD and ID and the associated comorbidities. Therefore, a French validation of both DiBAS-R (31–33) and the modified ADOS-2 version for older people with ASD and ID (34) is imperative. Moreover, a French psychometric validation of DAS (43) is needed. SCEB-A has been used in several studies to assess cognitive and socio-emotional development in children with ASD and other neurodevelopmental disorders and describe their level of development. Hence, future studies should test the validity of SCEB-A by comparing it with other developmental tests adapted to ages 4–24 months such as Scale of Emotional Development—Short (SED-S) (63, 64) and Bayley Scales of Infant and Toddler Development (65).

To specify the adaptive profiles of adults with ASD and severe ID, it would be interesting to compare them to adults with severe ID but without ASD.

### 6.2 Perform complementary studies on this clinical population

Complementary studies could be performed using our sample and data to explore the contribution of medical conditions to the adaptive developmental profile. This could be achieved by comparing adults with ASD and severe ID, with and without epilepsy, and those with and without behavioral and mental comorbidities.

Moreover, as the non-verbal developmental age of 18 months may be relevant for an ASD diagnosis (28), it would be interesting to compare the adaptive development of adults with ASD and ID and non-verbal ages inferior and superior to 18 months based on the non-verbal cognitive development domains assessed using SCEB-A.

Moreover, performing longitudinal studies regarding this group of adults and identifying their adaptive developmental trajectories (22) would be useful for evaluating the effects of their different clinical conditions.

### 6.3 Implications for interventions and coaching of adults with ASD and severe ID

This study’s implications on interventions and coaching of adults with ASD and severe ID should be considered in future studies. The goal should be to develop social communication programs and socialization training inspired by Early Start Denver Model (ESDM) (66) or Paediatric Autism Communication Therapy-Generalised (PACT-G) (67) (which is usually applied to children) and assess changes in adaptive development (22). Unfortunately, many—including professionals—consider that these very disabled adults cannot change, progress, or learn. However, an individualized program based on the adults’ cognitive, socio-emotional, and adaptive assessment showed that progress in the adaptive domain, although minimal, is possible (68).

## Data Availability

The raw data supporting the conclusions of this article will be made available by the authors, without undue reservation.

## Group member of Collaborative Working Group

Collaborative Authors: clinical psychologists and psychiatrists who examined the adults with ASD and severe ID 1BAUDOIN Céline, 1VAN TORNHOUT Marlène, 2,3TATON Romain (PhD), 4NÉZEREAU Célia (PhD), 5LEMPEREUR Aurélie, 6MENDES Elvira, 6TILLY LE CADRE Aurélie, 6Isabelle CARTEAU-MARTIN (PhD), 7MONTEIL Laurence, 7RECORDON-GABORIAUD Séverine (PhD), 8BARBILLAT-LEVASSEUR Mélanie, 9MOREL-KOHLMEYER Shasha (PhD), 9Emmanuelle HOUY-DURAND (Dr, PhD), 10LEGOUZOUGUEC Gildas, 11MAMOUNI Fatma (PhD), 12BREGER Coraline, 13DELÉPINE Emilie, 14HERNANDES PEQUENO Dionisia, 15FILIPOVA Marina (PhD), 16Aubane LAFLEUR, 16Claire DEGENNE (PhD). Collaborative Medico-Social Services 1Association Emmanuelle Châtelaillon-Plage (17), Maison d’Accueil Spécialisée, M.A.VIE, Impasse Clémenceau, 17340 Châtelaillon-Plage. 2Association Sésame Autisme Normandie, FAM « Le Roncier », 30, route du Roncier, 76890 Saint Victor l’Abbaye 3CRA Haute Normandie, Rouen Centre hospitalier du Rouvray 4 rue Paul Eluard – BP45 / 76301 Sotteville - Les - Rouen 4Cabinet de Psychologie ESPAS-Sup, 97, avenue Charles de Gaulle, 92200 Neuilly sur Seine 5Association Sésame Autisme Complexe Terres de Rouvre, 25, bis route d’Houppeville 76960 Notre Dame de Bondeville 6ADAPEI 37, 27 Rue des Ailes, ZA n°2 37210 Parçay-Meslay et Foyer de Vie La Bellangerie, ADAPEI 37, 37210 Vouvray 7APAJH 87, , MAS Aixe sur Vienne, 7 avenue François Mitterrand, 87700 Aixe sur Vienne 8Pôle d’appui et de ressources Autisme et TND de l’APAJH 86, 25 rue de Saint Nicolas, 86440 Migne Auxances 9Centre de Ressources Autisme et Pôle de psychiatrie. Centre Universitaire de Pédopsychiatrie, CHRU Bretonneau, 2, boulevard Tonnellé 37000 Tours 10APAJH 45, EAM Léonard de Vinci, 67, chemin des sablons, 45770 Sarran 11Le Silence des Justes, 18, rue Goubet 75019 Paris 12 Association SAUGE, FAM La Lendemaine, rue des Fonds d’Armenon, 91470 Les Molières 13ADAPEI 44, FAM Autisme Diapason, Moulin des Landes 44119 Grandchamp-des-Fontaines 14Maison Perce-Neige de Truyes Le Hameau 5, route de Cigogné 37320 Truyes 15ADAPEI 28, Les Papillons Blancs d'Eure et Loir, MAS Danois SAVS - Service d'Accompagnement à la Vie Sociale du Dunois, 7 rue Anatole France 28200 Châteaudun 16APAJH 33, MAS Le Barail, 43, avenue Jean Monnet 33700 Mérignac.
