# Corrigendum: Profile and development of adaptive behavior in adults with autism spectrum disorder and severe intellectual disability

**DOI:** 10.3389/fpsyt.2025.1579158

**Published:** 2025-03-26

**Authors:** Jean-Louis Adrien, Romuald Blanc, Eric Thiébaut

**Affiliations:** ^1^ Laboratory of Psychopathology and Health Processes (UR4057), Institute of Psychology, University Paris City, Paris, France; ^2^ Laboratoire Lorrain de Psychologie et Neurosciences de la Dynamique des Comportements, Université de Lorraine, Nancy, Lorraine, France

**Keywords:** autism spectrum disorder, severe intellectual disability, Vineland-II, profiles of socio-adaptive development, heterogeneity, autonomy, Socio-Emotional and Cognitive Evaluation Battery-Adult - SCEB-A

In the published article, there was an error in the **Abstract**, *Results*. This sentence previously stated:

“By contrast, the highest levels corresponded to Writing and Personal and Domestic Autonomy.”

The corrected sentence appears below:

“By contrast, the highest levels corresponded to Personal and Domestic Autonomy.”

In the published article, there was an error in **Figure 2** as published. The mean value of Communication DAE was recalculated. The corrected **Figure 2** and its caption appear below: 

**Figure 2:**
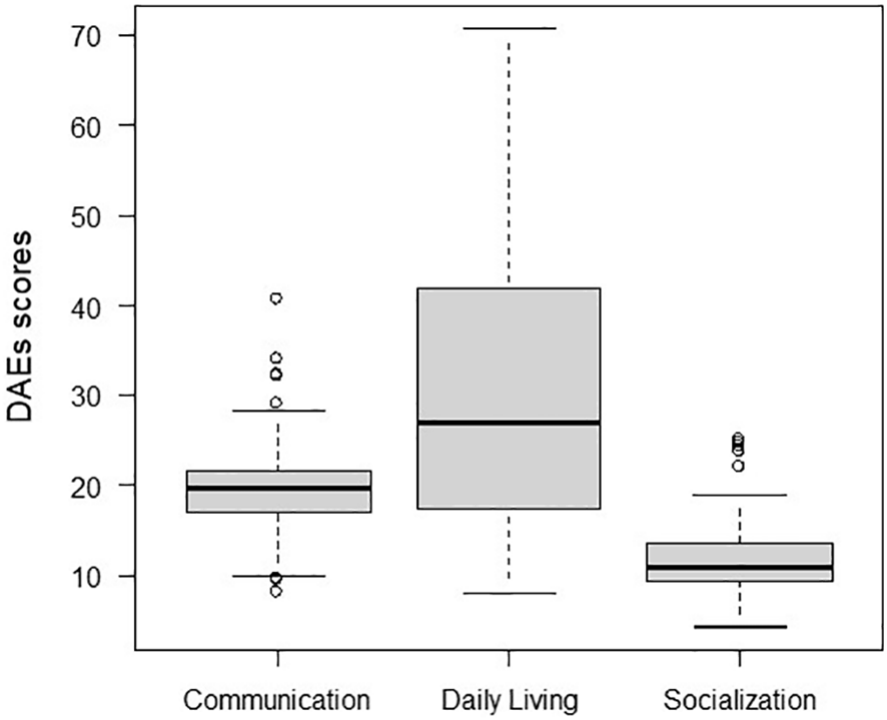
Profile of the median development ages equivalent (DAE) and interquartile range of the three domains of socio-adaptive development.

In the published article, there were errors in **Table 2** as published. The values of 'VABS-II – Global DAE' and 'VABS-II - DAE Communication' were recalculated. 'VABS-II—Standard score Communication, *Scale score Written*' and 'VABS-II—DAE Communication, *DAE Written*' were removed. The caption previously stated:

**Table 2:** Values of mean (M), median (Md), Standard Deviation (SD), interquartile range (IQR), minimum and maximum of standard scores of the 3 domains and scale scores of the 8 subdomains of the 69 adults (VABS-II).

	M	Md	SD	IQR	Min	Max
VABS-II – Standard score Communication	20	20	0	0	20	20
1. Scale score Receptive	1.1	1	.6	0	1	6
2. Scale score Expressive	1	1	0	0	1	1
VABS-II - Standard score Daily Living	20.2	20	1.3	0	20	29
1. Scale score Personal Autonomy	1.04	1	.27	0	1	3
2. Scale score Domestic Autonomy	1.6	1	1.3	1	1	7
3. Scale score Community Autonomy	1	1	0	0	1	1
VABS-II - Standard score Socialization	20	20	0	0	20	20
1. Scale score Interpersonal Relationships	1	1	0	0	1	1
2. Scale score Play/Leisure	1	1	0	0	1	1
3. Scale score Adaptation	1	1	0	0	1	1
VABS-II – Global DAE	19.5	17.6	8.2	11,8	8.4	42.9
VABS-II - DAE Communication	14.4	12.5	6.9	5	8	50
1. DAE Receptive	15.9	16.0	7.0	6.0	8.0	39
2. DAE Expressive	12.9	10.0	8.7	6.0	7.0	61
VABS-II - DAE Daily Living	31.1	27.0	16.6	24.7	8.0	70.7
1. DAE Personal Autonomy	33.3	31.0	17.1	21.0	8.0	107
2. DAE Domestic Autonomy	40.9	27.0	32.4	50.0	5.9	127
3. DAE Community Autonomy	19.2	12.0	12.6	21.0	8.0	57
VABS-II - DAE Socialization	12.3	11.0	4.9	4.3	4.3	25
1. DAE Interpersonal Relationships	10.2	8.0	4.1	2.0	3.0	27
2. DAE Play/Leisure	11.6	8.0	6.8	4.0	8.0	41
3. DAE Adaptation	16.1	13.0	6.9	8.0	8.0	37

Values of the Development ages equivalent (DAE) of 3 domains of adaptive functioning and the 8 subdomains of the 69 adults (VABS-II). VABS, Vineland Adaptive Behavior Scale.

In gray: Standard and DAE scores of three domain.

“Values of mean (M), median (Md), Standard Deviation (SD), interquartile range (IQR), minimum and maximum of standard scores of the 3 domains and scale scores of the 9 subdomains of the 69 adults (VABS-II). Values of the Development ages equivalent (DAE) of 3 domains of adaptive functioning and the 9 subdomains of the 69 adults (VABS-II).”

The corrected **Table 2** and its caption appear below:

In the published article, there was an error in **Table 3** as published. The correlations between 'DAE Communication subdomains, *Written DAE, Expressive DAE and Receptive DAE‘* were removed. The corrected **Table 3** and its caption appear below:

**Table 3:** Testing for pairwise differences in median development ages equivalent of domains and sub-domains scores Vineland-II of the 69 adults.

Pairwise comparison			*χ*² (Durbin-Conover)	*p*
Three domains of adaptive functioning				
DAE Communication	–	DEA Daily Living	15.3	< .001
DAE Communication	–	DEA Socialization	2.58	.011
DAE Daily Living	–	DEA Socialization	17.88	< .001
DAE Communication subdomains				
Receptive	–	Expressive	4.8	< .001
DAE Daily Living subdomains				
Personal	–	Community	8.06	< .001
Personal	–	Domestic	0.22	0.824
Community	–	Domestic	8.28	< .001
DAE Socialization subdomains				
Interpersonal Relationships	–	Play/Leisure	1.93	0.055
Interpersonal Relationships	–	Adaptation	11.16	< .001
Play/Leisure	–	Adaptation	9.23	< .001

In the published article, there was an error in **3 Results**, *3.2 Adaptive functioning developmental profile*, Paragraph 1. The section previously stated:

“Therefore, because this work was concerned with adaptive development, only DAE values were considered for the statistical analysis. Moreover, as the VABS-II norms do not indicate DAEs for Communication, Daily Living, and Socialization domains, the DAEs calculated corresponded to the median and mean values of their three subdomains.

The corrected section appears below:

“Therefore, because this work was concerned with adaptive development, only DAE values were considered for the statistical analysis, excepted for Writing not interpretable in this study. Moreover, as the VABS-II norms do not indicate DAEs for Communication, Daily Living, and Socialization domains, the DAEs calculated corresponded to the median and mean values of their three subdomains (only two for Communication: Receptive and Expressive).”

In the published article, there was an error in **3 Results**, *3.3 Adaptive functioning profile by domains and subdomains*, Paragraph 1. This sentence previously stated:

“The values of DAE of three domains of adaptive functioning and the nine subdomains (VABS-II) of 69 adults are presented in **Table 2** (data were missing for two participants).”

The corrected sentence appears below:

“The values of standard scores, scale scores and DAE of three domains of adaptive functioning and the eight subdomains of 69 adults are presented in **Table 2** (data were missing for two participants).”

In the published article, there was an error in **3 Results**, *3.3.1, The three adaptive functioning domains*, Paragraph 4. This sentence previously stated:

“Comparisons of the DAEs for Communication, Daily Living, and Socialization were performed using nonparametric tests suitable for repeated measurements (*χ²* Friedman (2, *N* = 69) = 85.8, *p* <.001, *w* = 0.62). Multiple comparisons were followed by analytical comparisons [34] (**Table 2**).”

The corrected sentence appears below:

“Comparisons of the DAEs for Communication, Daily Living, and Socialization were performed using nonparametric tests suitable for repeated measurements (*χ²* Friedman (2, *N* = 69) = 101, *p* <.001, *w* = 0.73). Multiple comparisons were followed by analytical comparisons [34] (**Table 3**).”

In the published article, there were errors in **3 Results**, *3 Adaptive functioning profile by domains and subdomains.* The subheading 3.3.2 was incorrectly given as “*3.3.2 The nine adaptive domains*”. The correct subheading is “*3.3.2 The eight adaptive domains*”. There was also an error in Paragraph 2, which previously stated:

“For Communication (Multiple comparisons: *χ²* Friedman (2, *N* = 69) = 60.3, *p* <.001, *w* = 0.44) (**Table 2**), there were significant differences between the values of the Expressive, Receptive, and Written DAEs, with those of the Written subdomain (DAE = 37 months) being more developed than the two others.”

The corrected sentence appears below:

“For Communication (Multiple comparisons: *χ²* Friedman (2, *N* = 69) = 60.3, *p* <.001, *w* = 0.44) (**Table 3**), there were significant differences between the values of the Expressive and Receptive DAEs with those of receptive subdomain being more developed.”

In the published article, there was an error in **3 Results**, *3.4 Relationships between socio-adaptive development and autistic symptomatology*, Paragraph 2. This sentence previously stated:

“The severity of autistic symptomatology was significantly correlated with a low overall socio-adaptive level, a low level of autonomy in daily living (particularly the levels of development of domestic autonomy and autonomy in the community), and a low level of expressive communication”.

The corrected sentence appears below:

“The severity of autistic symptomatology was significantly correlated with a low overall socio-adaptive level, a low level of autonomy in daily living (particularly the levels of development of domestic autonomy and autonomy in the community), and a low level of expressive and receptive communication”.

In the published article, there was an error in **5 Discussion**, Paragraph 4. This sentence previously stated:

“Results show evidence that the mean of the median DAE of socio-adaptive development (21.1 months) was slightly higher than that of cognitive and socio-emotional development (12 to 15 months), contrary to what was noted previously (17, 18).”

The corrected sentence appears below:

“Results show evidence the median of the global DAE of socio-adaptive development (17.6 months) was slightly higher than that of cognitive and socio-emotional development (12 to 15 months), contrary to what was noted previously (17, 18).”

In the published article, there was an error in **5 Discussion**, Paragraph 5. This sentence previously stated:

“The median DAEs in the three domains of socio-adaptive development of these adults were as follows: the DAE of Communication was 19.7 months, the DAE of Daily Living was 27 months, and the DAE of Socialization was 11 months.”

The corrected sentence appears below:

“The median DAEs in the three domains of socio-adaptive development of these adults were as follows: the DAE of Communication was 12.5 months, the DAE of Daily Living was 27 months, and the DAE of Socialization was 11 months.”

The authors apologize for these errors and state that they do not change the scientific conclusions of the article in any way. The original article has been updated.

